# Scraps

**Published:** 1895-12

**Authors:** 


### SCRAPS

PICKED UP BY THE ASSISTANT-EDITOR.

---

**Physiology.**—The following composition by a twelve-year-old schoolboy was the cause of his being recommended to take a special course in physiology the next term. The theme given him was "Breath." "Breath is made of air. We always breathe with our lungs, and sometimes with our livers, except at night, when our breath keeps life going through our noses while we are asleep. If it wasn't for our breath, we should die whenever we slept. Boys that stay in a room all day should not breathe; they should wait till they get outdoors. For a lot of boys staying in a room make carbonicide; and carbonicide is more poisonous than mad dogs, though not just the same way. It does not bite; but that's no matter as long as it kills you."

**Medical Men and Medicine Men.**—In a paper on this topic, Dr. E. J. Overend, of San Francisco, says: "There is a way of educating the public to a decent understanding of the limitations of medical science. It consists in telling patients what we do not know as well as what we know. They should be taught that medicine is made up of a number of sciences, more or less exact, the less exact of which is therapeutics, the very one by which they judge the whole. When that time arrives the patient will be satisfied with the scientific regimen laid down by the physician, and not look for a 'bottle of bitterness' as an indispensable adjunct. Drug mania has at present full sway in the profession, constituting another reason why the average doctor is in some danger of being called a 'medicine man.' The surgeons, as a rule, in avoiding Scylla, are drawn into Charybdis, they are open to the objection that they are afflicted with *pruritus secandi*. The profession needs a robust scepticism for its protection, not only from manufacturing pharmacists, but even from prominent members, who advocate with persuasive pens novel but worthless methods."—*Occidental Medical Times.*

**Clinical Records** (13).—Un jeune Esculape, fraîchement nanti de son diplôme, est d'une extrême timidité. Chaque fois qu'il est appelé près d'une jeune femme, il se trouble, tâte le pouls en se tenant à distance et se trouve dérouté pour un rien.

Tout récemment il est demandé près d'une dame souffrant d'accidents abdominaux. Bien timidement il s' approche et, sans soulever la couverture, il glisse sa main dans le lit, pour pratiquer la palpation abdominale. Le ventre est chaud, gonflé, non douloureux à la pression. La fluctuation est manifeste. Décidément il s'agit d'une tumeur liquide, vraisemblablement d'un kyste ovarien. Notre jeune confrère hasarde timidement le diagnostic tumeur et demande une consultation. En attendant il prescrit un traitement inoffensif. Il allait se retirer lorsque la malade le rappelle et lui dit: "Mon cher docteur, j'espère qu'après avoir prescrit un traitement pour mon sac en caoutchouc, vous allez en prescrire un pour moi."

Notre jeune novice avait pris une vessie pleine d'eau chaude pour le ventre de sa cliente.—*La Revue Médicale.*

**Medical Missions.**—Our attention is now painfully directed towards China. Mr. D. Duncan Main, the Medical Officer of the Hospital at Hang-Chow, where 11,865 new dispensary patients were treated in 1894, says:—

"Most of the diseases we have to treat are due to insufficient food, bad food, uncooked food, insufficient clothing, malaria, and dirt. Dirt certainly brings us thousands every year. There is always an epidemic of hydrophobia, in its literal meaning, in China. Many of the patients are very poor, and we must draw a veil of charity over a great deal, because many of the poor creatures come in their wardrobe, which can never be washed, because there is nothing to take its place. The superstition we have to deal with is even worse than their physical condition. I must confess it is not always easy in dispensary work to be patient and perfectly just in thought and feeling with superstitious patients, who seem to think, by virtue of their disease, they have a right to demand a heap of time and attention and an extra supply of (*tin hao tih yah*) very good medicine. It takes a great deal of grace to keep smiling and self-forgetful, especially when one's own vitality is at a low ebb, and nerves are all unstrung through overwork and constant contact with the diseased and dying. During hot-house, high-pressure days, when the thermometer is over 100 deg. in the shade and the atmosphere of the waiting-room is thick and very much tarnished, dispensary work is most trying; still, it affords many opportunities of triumphing over self!

"It is in the wards that the medical missionary is brought into contact with the patients under the most favourable conditions for making an impression, and convincing the patients that he is practically interested in them, both physically and spiritually. On the other hand, the patients are able to study our aims and objects, and to *see* the practice of Christianity, as well as *hear* the theory.

"In the autumn a mandarin, who had been in a private ward for three weeks undergoing treatment, handed me a packet containing $100 as a small donation to help on our good work. He said he had been watching me during his stay, and had wondered very much why I never asked him for money! He also said he knew Chinamen who had money and did deeds of kindness for their own people, but he did not know any of his own countrymen who left home and friends and went to a foreign country to carry on a work of charity. There can be no doubt that there are many who believe in a charity which shows itself in practical benevolence and treatment of disease. A Chinaman has faith in anything that will prolong his days, and he certainly looks with respect and confidence upon the man who can heal disease."—*Proceedings of the Church Missionary Society.* 1895.

**Medical Philology** (XVI.)—The use of "child" either as a transitive or intransitive verb, and of "childing" as a verbial substantive or a participial adjective, has long dropped out of ordinary English talk. But at one time they were recognised forms. The *Catholicon* (1483) has "to Childe; *parturire, eniti, fetare, parere, profundere.*" The *Promptorium* (*c.* 1440) has "Chyyldyn̄, or bryngyn̄ furthe chylde *Pario.* Chyldynge, or woman wythe chylde. *Pregnans.*" Mr. Way, Mr. Herrtage, and the *New English Dictionary* give several illustrations of these early forms. Some of these I arrange in chronological order, but the first of the illustrations is not to be found amongst them. "Cildiungwif," as the equivalent of "puerpera," occurs in Archbishop Alfric's Vocabulary, a work of the tenth century (Wright's *Vocab.*, 108, 17). Orm, the writer of the series of Homilies known as the *Ormulum* (*c.* 1200), says: "þe shall Elysabæþ þin wif an sune childenn" (ed. Holt, 156). Describing the visit of the Blessed Virgin Mary to her kinswoman Elizabeth, the *Cursor Mundi* (*c.* 1280) has:

"þar duelld vr lauedi wit hir nece,

Til ion was born, a wel godd pece,

At hir childing hir was helpand"

E.E.T.S., ed. Morris, p. 634, ll. 11057–9.

In *Arthour and Merlin* (*c.* 1330) is the passage: "Sche childed a selcouthe grome" (Abbotsford Club ed., 978); or, in modern English, "She brought forth a wonderful boy." I have not been able to refer to this work, but the passage, I presume, alludes to the birth of Merlin. The *Ayenbite of Inwyt*, or Remorse of Conscience (1340), has: "þe wyfman lyþ a chi[l]dbedde oþer nyeȝ to childi" (E.E.T.S., ed. Morris, p. 224). Maundevile (1356), giving the record of the Incarnation according to the Saracens, says that Mary "childed undre a Palme Tree" (ed. Halliwell, p. 133). Wyclif's reading of Genesis xix. 37, 38 (1382) is: "The more douȝtir childide a sone, and clepide his name Moab . . . and the lesse douȝtir a son, and clepide his name Amon." In the 15th century translation of Higden's *Polychronicon* mention is made of a woman who "hadde vij. childer at oon childinge" (Rolls Series, ed. Lumby, I, 205). In the story, "A Prophecy Fulfilled," in the *Gesta Romanorum* (*c.* 1440), Dolfinus, wishing to destroy the forester's new-born babe that he had been told in a dream would succeed him, sent messengers to the house of the forester, telling them to take "the little Infaunt, that his wyf this nyght chylded" (E.E.T.S., ed. Herrtage, p. 209). In *Knight de la Tour Landry* (*a.* 1450) is the statement that "whanne she hadde childed she thanked God" (E.E.T.S., ed. Wright, 108). The *Medulla* (1468) has "*Pario.* To chyldyn. *Parturio.* To ympyn, beryn, or chyldyn." Latimer (1549), in his Second Sermon before Edward VI., in order to show that "it is wisdom and godly knowledge that causeth a king to be feared," introduced the incident of the appeal which the two mothers made to Solomon, and said "within ii dayes they chylded both" (ed. Arber, 71). Spenser has "chylded" (*Faerie Queene*, VI. xii. 17), and Heywood (1611), in the *Golden Age*, IV. i., says, "The Queene shall childe a daughter beautifull."

"Childing" as a participial adjective was very common and lasted much longer. In its figurative sense, it is familiar to readers of Shakspere, who will remember Titania's mention of "the childing autumn" (*Mid.-N.D.* II. i. 112). In this adjectival form it continued till the present century, and is even now often met with in botanical definitions.

